# Awareness of Common Cancer Risk Factors and Symptoms in Saudi Arabia: A Community-Based Study

**DOI:** 10.31557/APJCP.2021.22.6.1813

**Published:** 2021-06

**Authors:** Maaidah Algamdi, Analita Gonzales, Ehab Farah

**Affiliations:** 1 *Department of Nursing, Faculty of Applied Medical Sciences, Tabuk University, Saudi Arabia. *; 2 *Department of Statistics, Faculty of Science, Tabuk University, Saudi Arabia. *

**Keywords:** Cancer risk factors, cancer symptoms, community awareness, Saudi Arabia

## Abstract

**Objective:**

To assess the level of cancer awareness and the relationship between the awareness of common cancer symptoms and risk factors and the sample’s sociodemographic profile.

**Methods:**

A community-based cross-sectional study conducted in Tabuk city, a convenient sample of 675 participants completed a questionnaire about common factors causing cancer and symptoms related to cancer. Descriptive statistics and chi-squared analysis were used to measure sample characteristics and their association with knowledge of cancer risk factors and symptoms.

**Results:**

Level of education and family history were significantly related to awareness of cancer risk factors (P= 0.017) and (P= 0.048), respectively. Factors were significantly associated with awareness of cancer symptoms include Gender (P=0.000), nationality (P=0.013), and undergoing regular cancer screening tests (0.008). Internet was the primary source of information about cancer and related significantly to knowledge about cancer symptoms(P=0.000) and risk factors(P=0.00). More than half of the sample scored poorly for knowledge of both cancer risk factors (58.7%) and symptoms (66.2%) in terms of the overall level of cancer awareness. Smoking and heredity were the most identifiable cancer risk factors, and unexplained pain and weight loss were identified by the majority of study participants.

**Conclusion:**

The level of community knowledge requires further investigation, and more cancer awareness programs need to be conducted. People could be encouraged to use reliable sources of information to obtain accurate cancer information.

## Introduction

Cancer is the second leading cause of death worldwide and accounted for 9.6 million deaths in 2018 (WHO, 2018). Globally, the most prevalent cancers are lung, breast, colorectal, prostate, skin, and stomach cancer (National Cancer Institute, 2020). In Saudi Arabia, 24,485 new cases of cancer were detected in 2018 across all age groups and both sexes, including breast, colorectal, and thyroid cancer, leukemia, and non-Hodgkin’s lymphoma; these were also the top five cancers leading to mortality (International Agency for Research on Cancer, 2018). Despite the small number of cancer cases in Saudi Arabia compared with the rest of the world, the number of cases is expected to increase each year due to radical lifestyle changes and the Saudi population’s expected growth (Althubiti and Eldein, 2018). Therefore, understanding factors leading to cancer and warning symptoms of cancer could encourage individuals to pay more attention to their health concerns.

The National Cancer Institute (2015) reported risk factors of cancer including age, heredity, exposure to chemical substances, and ionizing or ultraviolet radiation. Limiting exposure to these factors can reduce the risk of some cancers (Ligibe, 2012). Unhealthy related behaviors and some environmental features pose potential risks that lead to cancer. As Kulhánová et al., (2019) indicate, lifestyle and environmental attributes associated with increased risks of cancer include smoking, obesity or being overweight, an unhealthy diet with insufficient consumption of fruit and vegetables, and a lack of physical activity. According to the National Cancer Institute (2019), cancer symptoms differ according to the organ involved; they include fever, nausea, weakness, and sudden body weight change (usually a decrease). In addition to variations in skin color (yellowness, redness, etc.), a visible lump or tumor may also form under the skin. Other symptoms include non-healing cuts, a constant cough, hoarseness, trouble swallowing, indigestion, and defecation difficulties (Saudi Ministry of Health, 2013). According to Quaife et al., (2014), people who properly recognize cancer symptoms increase their likelihood of seeking medical attention, receiving an earlier diagnosis, and surviving. However, a lack of knowledge of cancer-related symptoms has negative consequences for people’s health. Neal et al., (2015) stated that a delay in cancer diagnosis is a factor that negatively affects the outcome of treatment.

People modify their behavior based on their knowledge of cancer risk factors and seek medical advice if they are aware of warning symptoms. Smith et al., (2016) reported that cancer awareness has been considered a critical component of behaviour change and seeking help. Many studies have focused on the importance of increasing public awareness of cancer symptoms and risk factors and evaluating intentions for lifestyle changes (Smith et al., 2016; Waller, 2009). Smith et al., found differences in cancer awareness among visitors before and after their visit to a Cancer Awareness Roadshow, which ultimately improved their attitudes toward seeking help. Many studies in Saudi Arabia have observed a lack of cancer awareness (Ravichandran and Mohamed, 2011; Ibrahim, 2020). Public knowledge of cancer in terms of risk factors related to lifestyle or environment and unusual symptoms connected to cancer are considered essential elements of preventing cancer occurrences or decreasing the burden of treatment in late-stage cancer. Thus, it is crucial to assess the Saudi Arabian community’s awareness of cancer symptoms and risk factors, particularly for common cancers. This may bridge the gap in educating the public and possibly reduce the incidence of cancers by developing strategies to educate the community.

The aim of this study was to assess the level of cancer awareness and the relationship between the awareness of common cancer symptoms and risk factors and the sample’s sociodemographic profile. 

## Materials and Methods

A cross-sectional community-based study was conducted to explore the awareness of cancer risk factors and symptoms in Saudi Arabia. The sample was expected to reach 384 participants with a 95% confidence interval and 0.5 margins of error, (Polit and Beck, 2012). Considering Saudi culture sensitivity toward cancer, we assumed a 50% response rate and distributed 768 surveys to receive a sufficient number of responses (Ravichandran et al., 2011). The sample was selected based on the following inclusion criteria: proficient literacy and writing abilities, and aged 18 years or older. After obtaining ethical approval # TU-124-18-2020 from the University of Tabuk Local Research Ethics Committee, the study was conducted by distributing the surveys in multiple public areas, such as 2 parks, 3 restaurants, 3 coffee shops, and 3 malls. The purpose of the study was explained to potential participants, and informed consent was obtained. 

*Instrument*

The self-developed questionnaire consisted of sociodemographic characteristics and questions about the source of cancer information, undergoing cancer screening and performing self-examination, and symptoms and risk factors of cancer. Cancer symptoms and risk factors were selected based on common cancers in Saudi Arabia (International Agency for Research on Cancer, 2018). The questionnaire written in a simple language that can be easy for the participant to understand. We selected ageing and heredity as non-modifiable factors and smoking, unhealthy diet, red meat intake, obesity, and lack of exercise as modifiable factors (Ligibe, 2012; National Cancer Institute, 2019). The selected cancer symptoms were associated with the six most common cancers in Saudi Arabia and are easily noticed by individuals. These include unexplained pain, unexplained bleeding, persistent cough or painful breathing, unexplained weight loss, and unexplained loss of appetite (Saudi Ministry of Health, 2013). For evidence of reliability, Cronbach’s alpha for the 12-items questionnaire was 0.697 and 0.704 for standardized items. The level of knowledge was determined by adding up the score out of 7 for risk factors and 5 for symptoms, with a higher score indicating higher knowledge. The total score for risk factors ranged from 0–7, classified as 0–2 poor, 3–4 average, and 6–7 high level of awareness. For symptoms, the total score ranged from 0–5, categorised as 0–1, 2–3, and 4–5, indicating a poor, average, and high level of awareness respectively.

*Data Analysis*

The data were encoded in an excel sheet then transferred into SPSS software version 26 (IBM Corp, Armonk, New York). Descriptive statistics were generated for the sample description. Data were analyzed using the chi-square of independence to determine the associations between the sample’s sociodemographic characteristics and their level of awareness regarding risk factors and symptoms of cancer. A p-value of < 0.05 was considered significant. Symptoms and risk factors were ranked based on the frequency of participants’ selection of each item.

*Ethical Considerations*

Ethical approval was obtained before conducting the study. No vulnerable groups or sensitive issues were involved, and all participants consented before completing the survey. The participants were informed that participation was voluntary and that they could stop and withdraw without any consequences. Anonymity and confidentiality were maintained, and no personal identifier was required. Participant responses were kept confidential in a secured place. 

## Results

Out of 768 surveys, we received a total of 675 completed surveys, with a response rate of 87.8% and an attrition rate of 12.1%. Table 1 depicts the sociodemographic characteristics of the participants. 348 (51.6%) were female and 327 (48.4%) were male. The largest age group was 18 to 24 years old (42.8%), and the sample was dominated by Saudi participants (81%). Half of the participants (50.2%) were married, while just less than half were single (48.4%). Around half of the participants were employed (42.6%) while (20.1%) were unemployed. Nearly half held a bachelor’s degree (45%). The primary source of cancer information was from the internet (41.3%) and social media (18.8%), while 12.4% reported that they learned about cancer from awareness campaigns. The majority of the participants and their family members had never been diagnosed with cancer (79.1%), while (19.7%) of them had. Only 14.1% of participants had done regular screening tests for cancer; a substantial proportion (85.9%) had never done these tests. Similarly, most participants reported that they had never done a self-examination for cancer (83.7%), while just 16.3% had.

*Awareness of cancer risk factors*

No significant association between knowledge of cancer risk factors and sex, age, nationality, marital status, employment status, diagnosis of cancer, and undergoing routine cancer screening and self-examination. There was a significant association between knowledge of cancer risk factors and education level (P= 0.017). Participants with secondary and university level education exhibited poor risk factor knowledge (23.9 % and 27.3% respectively). Family history has a significant relationship with awareness of risk factors (P= 0.048). More than half of the participants (54.7 %) with no family history of cancer exhibited significantly lower risk factor knowledge than those with a family history of cancer (11%). A significant relationship was found between participants’ sources of information and awareness of cancer risk factors (P= 0.000). Around one thirds of participants (27.4%) selected internet as the main source of information about cancer risk factors and scored a poor level of knowledge. Overall awareness regarding cancer risk was significantly related to participants’ level of awareness (P=0.000). More than half of the sample (58.7%) had a low level of knowledge, and (34.1%) had an average level, and just 7.3% had a high level of awareness (Table 2).

*Awareness cancer symptoms*

There was no significant association between knowledge of cancer symptoms and age, marital status, level of education, employment status, family history, diagnosis of cancer, and undergoing regular self-examination. There was a significant association between knowledge of cancer symptoms and sex (P= 0.000). One third of participants were male (30.8%) scored poor symptoms knowledge compared to females (27.9%). Nationality has significant relationship with awareness of cancer symptoms (P= 0.013). Around half of the participants (48%) were Saudis exhibited significantly poorer knowledge of cancer symptoms than non-Saudis. Participants’ sources of information have a significant relationship with awareness of cancer symptoms (P = 0.000). highest percentage of participants (23.6%) selected internet as their primary source of cancer information scored poor on their level of knowledge of symptoms. Undergoing regular cancer screening tests has a significant association with knowledge about cancer symptoms (P =0.008). Half of participants (50.7%) were never undergone regular cancer screening test scored a poor level of symptom awareness. Overall awareness regarding cancer symptoms was significantly related to participants’ level of awareness (P =0.000). More than half of the sample (66.2%) had a poor level of cancer symptom awareness. 25% of the sample had average knowledge, and only 8.1% exhibited a high level of awareness (Table 3).

*Ranking risk factors and symptoms*

We ranked risk factors and symptoms from highest to lowest frequency based on participants’ selection. Regarding risk factors, smoking was the most identified factor (known by 71% of the sample), followed by an unhealthy diet (37.2%), heredity (35.3%), a lack of physical activity (23.4%), obesity (22.1%), excessive consumption of red meat (13.9%), and ageing (10.4%). For symptoms, unexplained pain was selected by more than half of the sample (51.0%), followed by unexplained weight loss (36.4%), unexplained loss of appetite (30.7%), unexplained bleeding (21.2%), and persistent cough or painful breathing (16%).

**Table 1:** Sociodemographic Characteristics of the Studied Sample from Tabuk City, Saudi Arabia (N=675)

Sociodemographic characteristics		n (%)
Sex	Female	348 (51.6)
	Male	327 (48.4)
Age	18–24	247 (36.6)
	25–34	165 (24.4)
	35–44	142 (21)
	> 45	79 (11.7)
	Missing	42 (6.2)
	Saudi	547 (81)
Nationality	Non-Saudi	128 (19)
Marital status	Married	339 (50.2)
	Single	327 (48.4)
	Divorced	6 (.9)
	Widowed	3 (.4)
Employment status	Employed	287 (42.6)
	Student	252 (37.3)
	Not employed	136 (20.1)
Level of education	Primary	31 (4.6)
	Intermediate	73 (10.8)
	High school	239 (35.4)
	Bachelor’s degree	304 (45)
	Other	28 (4.1)
Source of information	Family	61 (9.0)
about cancer	Internet	279 (41.3)
	Television	66 (9.8)
	Social media	127 (18.8)
	Awareness campaigns	84 (12.4)
	Other	58 (8.6)
Have you been diagnosed with cancer?	Yes	21 (3.1)
	No	651 (96.4)
	Prefer not to answer	3 (.4)
Do you have a family member who has been diagnosed with cancer?	Yes	133 (19.7)
	No	534 (79.1)
	Prefer not to answer	8 (1.2)
Have you had regular screening tests for cancer?	Yes	95 (14.1)
	No	580 (85.9)
Have you performed self-examinations for cancer (breasts or testicles)?	Yes	110 (16.3)
	No	565 (83.7)

**Table 2:** Association between Awareness of Risk Factors with Sociodemographic Characteristics (N=675)

Sociodemographic characteristics		Poor risk factor knowledge		Average risk factor knowledge		High risk factor knowledge		P-value
		n	%	n	%	n	%	
Sex	Male	222	32.9	81	12	24	3.6	0.619
	Female	225	33.3	92	13.6	31	4.6	
Age	24–18	149	22.1	73	10.8	25	3.7	0.199
	34–25	106	15.7	46	6.8	13	1.9	
	44–35	101	15	30	4.4	11	1.6	
	≥ 45	60	8.9	16	2.4	3	0.4	
Nationality	Saudi	363	53.8	143	21.2	41	6.1	0.404
	Non-Saudi	84	12.4	30	4.4	14	2.1	
Marital status	Married	237	35.1	80	11.9	22	3.3	0.289
	Single	203	30.1	92	13.2	32	4.7	
	Widowed	3	0.4	0	0	0	0	
	Divorced	4	66.7	1	16.7	1	16.7	
Level of education	Primary	26	3.9	2	0.3	3	0.4	0.017*
	Intermediate	53	7.9	17	2.5	3	0.4	
	Secondary	161	23.9	64	9.5	14	2.1	
	University	184	27.3	87	12.9	33	4.9	
	Other	23	3.4	3	0.4	2	0.3	
Employment status	Employed	194	28.7	70	10.4	23	3.4	0.688
	Student	160	23.7	68	10.1	24	3.6	
	Not Employed	93	13.8	35	5.2	8	1.2	
Family history of cancer	No	369	54.7	125	18.5	40	5.9	0.048*
	Yes	74	11	45	6.7	14	2.1	
	No answer	4	0.6	3	0.4	1	0.1	
Diagnosed with cancer	No	432	64	168	24.9	51	7.6	0.366
	Yes	14	2.1	4	0.6	3	0.4	
	No answer	1	0.1	1	0.1	1	0.1	
Source of information about cancer	Family	42	6.2	14	2.1	5	0.7	0.000*
	Internet	185	27.4	76	11.3	18	2.7	
	Television	52	7.7	12	1.8	2	0.3	
	Social media	85	12.6	37	5.5	5	0.7	
	Campaigns	47	7	25	3.7	12	1.8	
	Other	36	5.3	9	1.3	13	1.9	
Regular cancer screening tests	Yes	63	9.3	21	3.1	11	1.6	0.344
	No	384	56.9	152	22.5	44	6.5	
Regular self-examinations	Yes	62	9.2	36	5.3	12	1.8	0.057
	No	385	57	137	20.3	43	6.4	
Overall awareness		396	58.7	230	34.1	49	7.3	0.000*

Note: P-value, level of significance; *, Significance level < 0.05

**Table 3:** Association between Awareness of Symptoms with Sociodemographic Characteristics (N=675)

Sociodemographic characteristics		Poor symptom knowledge		Average symptom knowledge		High symptom knowledge		P-value
		n	%	n	%	n	%	
Sex	Male	208	30.8	86	12.7	33	10.1	0.000*
	Female	188	27.9	144	21.3	16	2.4	
Age	24–18	135	20.0	92	13.6	20	0.3	0.473
	34–25	103	15.3	55	8.1	7	1.0	
	44–35	84	12.4	46	6.8	12	1.8	
	≥ 45	50	7.4	21	3.1	8	1.2	
Nationality	Saudi	324	48.0	191	28.3	32	4.7	0.013*
	Non-Saudi	72	10.7	39	5.8	17	2.5	
Marital status	Married	205	30.4	108	16.0	26	3.9	0.891
	Single	185	27.4	119	17.6	23	3.4	
	Widowed	2	0.3	1	0.1	0	0.0	
	Divorced	4	0.6	2	0.3	0	0.0	
Level of education	Primary	22	3.3	8	1.2	1	0.1	0.24
	Intermediate	42	6.2	29	4.3	2	0.3	
	Secondary	146	21.6	73	10.8	20	3.0	
	University	167	24.2	114	16.9	23	3.4	
	Other	19	2.8	6	0.9	3	0.4	
Employment status	Employed	179	26.5	83	12.3	25	3.7	0.052
	Student	143	21.2	90	13.3	19	2.8	
	Not Employed	74	11.0	57	8.4	5	0.7	
Family history of cancer	No	328	48.6	168	24.9	38	5.6	0.61
	Yes	65	9.6	58	8.6	10	1.5	
	No answer	3	0.4	4	0.6	1	0.1	
Diagnosed with cancer	No	382	56.6	223	33	46	6.8	0.397
	Yes	12	1.8	7	1.0	2	0.3	
	No answer	2	0.3	0	0.0	1	0.1	
Source of information about cancer	Family	42	6.2	18	2.7	1	0.1	0.000*
	Internet	159	23.6	101	15	19	2.8	
	Television	51	7.6	11	1.6	4	0.6	
	Social media	68	10.1	53	7.9	6	0.9	
	Campaigns	41	6.1	35	5.2	8	1.2	
	Other	35	5.2	12	1.8	11	1.6	
Regular screening tests	Yes	54	8.0	27	4.0	14	2.1	.008*
	No	342	50.7	203	30.1	35	5.2	
Regular self-examinations	Yes	54	8.0	48	7.1	8	1.2	0.061
	No	342	50.7	182	27.0	41	6.1	
Overall awareness		447	66.2	173	25.6	55	8.1	0.000*

Note: P-value, level of significance; *Significance level < 0.05

## Discussion

This study aimed to assess community knowledge about cancer in terms of risk factors and symptoms. The primary sources of cancer information were the internet and social media. This finding is supported by Ibrahim et al., (2020), who mentioned that people use the internet as their source of cancer information. Awareness campaigns were identified as a source of information by only 12.4% of the sample. This is alarming as it suggests that the effectiveness of cancer awareness campaigns is lower than expected. Due to the radical changes in people’s lifestyles, Al-Maweri et al., (2015) recommend using mass media to increase public awareness of cancer. Using media to spread cancer information would likely have a more significant impact on future generations and could be more cost-effective (Worthington et al., 2020). Screening for cancer or performing self-examinations were not considered by most of the studied sample (85.9%). This finding is supported by AlAyadhi et al., (2020), who demonstrated that most people never undergo screening.

Level of education exhibited a significant relationship with the awareness of cancer risk factors. This finding is supported by Ravichandran et al., (2010) study, which revealed that level of education was associated with the number of risk factors recognised by participants; participants who had primary, secondary, or higher education recognised significantly more risk factors than those with no formal education. Furthermore, Al-Azri et al., (2014) found a significant association between participants’ responses and their education level. The higher their education, the more likely they were to identify cancer risk factors. While in the current study, participants with higher education, opposite to the previous studies findings, scored low risk factors knowledge. The present study observed a significant relationship between a family history of cancer and risk factor awareness; the same finding was reported by Poudel and Naomi (2017). Based on the current findings, internet was the primary health educator for most participants in terms of risk factors awareness, although the low knowledge level of internet users. We can infer that internet have a leading role in educating the community. As mentioned in Nazir and Soroya (2021) findings, the internet is primary source of health information of most of their studied sample. 

Regarding gender, Ravichandran et al., (2010), Qassim et al., (2018), and Feizi et al., (2011) demonstrated that females have more cancer-related knowledge than males. This study had similar findings: males earned lower scores than females, indicating fewer symptoms and risk factors. Women, who may be at greater risk of getting cancer than men due to regular hormonal changes, are arguably more likely to learn about cancer symptoms (Dorak and Karpuzoglu, 2012). Regarding nationality, Saudis, as reported by many studies, have low cancer knowledge (Ravichandran et al., 2010; Feizi et al., 2011). Also, in the current study, many Saudis scored lower in terms of knowledge of cancer symptoms relative to non-Saudis. Nazir and Soroya (2021) reported that internet was primary source of seeking health information, and similar to the current findings, internet was selected by most participants; however, their level of awareness was low. Furthermore, the type and nature of websites might convey inadequate information about cancer risk factors and symptoms. 

Our study showed poor overall knowledge of both symptoms and risk factors of cancer, a finding supported by Ibrahim et al., (2020) that revealed a lack of public awareness of cancer in Saudi Arabia. Therefore, there is an urgent need to increase cancer awareness among the Saudi population. The present study revealed that smoking is the most recognised risk factor for cancer, selected by 71% of the sample. This is supported by Al-Azri et al., (2014) and Schliemann et al., (2020) which mentioned smoking as the highest risk factor for cancer. The public generally has a high level of knowledge of the dangers of smoking due to ongoing campaigns to raise awareness about smoking’s consequences on people’s health (Van Mourik, 2020). An unhealthy diet was the second most identified risk factor (37.2%) in this study. This is supported by Sharma et al. (2019) which reported that half of participants recognised lifestyle-related risk factors such as drinking more than one unit of alcohol or eating less than five portions of fruits and vegetables per day, and eating red or processed meat once a day or more; these are all related to an unhealthy diet. Alyami et al., (2020) reported that smoking and lifestyle factors were the most prevalent factors selected by their sample. In the present study, around a third of the sample selected heredity (35.5%) as a risk factor. Al-Azri et al., (2014) study also reported family history as one of the risk factors as cancer could be considered hereditary. A lack of physical activity and obesity were the fourth and fifth most recognised risk factors in this study. In studies by Ravichandran et al., (2010) and Sharma et al., (2019), being obese and being physically inactive were also identified as risk factors by the participants. Ageing was selected as a risk factor by 10.4% of our sample, while around 28% of respondents (133 participants) in Sharma et al., (2019) study recognised older age as a risk factor. 

In the current study, unexplained pain was the most identified symptom for cancer symptoms, followed by unexplained weight loss, loss of appetite, bleeding, and persistent cough and painful breathing. More than half of the studied sample linked cancer with pain, in line with the findings of Schliemann et al., (2020). Unexplained weight loss and appetite loss were selected by around a third of the studied sample. This is supported by a study where around a third of participants saw unexplained weight loss as an early warning sign of cancer (Schliemann et al., 2020). Only 16% of our sample selected persistent cough or painful breathing as a cancer symptom, while Sharma et al., (2019) study revealed that around half of their respondents (228 participants) believed that a persistent cough or a hoarse voice could be early warning signs of cancer. 

The study’s conclusion increasing the level of knowledge of the community requires further efforts and investigation. People should be encouraged to utilize appropriate and accurate sources of health related information and undergo screening tests when required. Increasing the participants’ level of awareness would involve tailoring educational programs to improve their knowledge about cancer. This knowledge could then be reflected in the community’s behaviour in the kingdom of Saudi Arabia. 

## Author Contribution Statement

Dr.Algamdi and Dr.Gonzales in conceptualization and academic writing, Dr.Farah in statistical analysis.

## Declarations

This study received ethical approval # TU-124-18-2020 from the University of Tabuk Local Research Ethics Committee.
